# Integrated systems biology reveals an 8-gene signature predicting early-stage lung adenocarcinoma progression and patient survival

**DOI:** 10.1038/s41598-025-18567-w

**Published:** 2025-10-06

**Authors:** Corey D. Young, Kaylin M. Carey, Courtney D. Dill, Sha’Kayla K. Nunez, Ti’ara L. Griffen, Eric B. Dammer, James W. Lillard

**Affiliations:** 1 Division of Cancer Epidemiology and Genetics, Department of Health and Human Services, National Cancer Institute, National Institutes of Health, Bethesda, MD USA; 2 Department of Microbiology, Biochemistry, and Immunology, Morehouse School of Medicine, 720 Westview Dr SW, HG 341B, Atlanta, GA 30310 USA; 3 Center for Neurodegenerative Disease, Emory University School of Medicine, Atlanta, GA 30322 USA; 4 Department of Biochemistry, Emory University School of Medicine, Atlanta, GA 30322 USA; 5 Genentech, 1 DNA Way, South San Francisco, CA 94080 USA

**Keywords:** Non-small cell lung cancer, Early-stage, Biomarkers, RNA sequencing, Survival, Prognostic signatures, Cancer, Cancer genomics, Lung cancer, Tumour biomarkers

## Abstract

A comprehensive analysis of the transcriptomic landscape in early-stage LUAD (stages 1 and 2) can identify robust survival and staging predictors. Using a systems-biology approach with TCGA LUAD transcriptomic data, we identified 18 co-expression modules, 11 correlated with staging and 7 with survival. Five survival- and staging-associated (SAS) modules (M1, M3, M6, M9, and M16) distinguished patients with differing survival curves based on module eigengene expression. Anti-correlated SAS-modules (M3 and M6) exhibited the strongest associations with OS and staging. Key differentially-expressed hub genes from M3 and M6 were nominated as prognostic markers through iterative combinatorial ROC analysis. Survival prediction was refined by testing equal-weight gene ratios with opposing correlations to survival, revealing additive or synergistic predictive value. The top-performing ratio, ATP6V0E1 + SVBP + HSDL1 + UBTD1/GNPNAT1 + XRCC2 + TFAP2A + PPP1R13L, achieved an average AUC of 75.5% across three timepoints (12 months, 18 months, and 3 years). Comparison with three established LUAD prognostic signatures (Shedden, Soltis, and Song) within the TCGA network showed that our 8-gene signature had comparable or superior predictive power while maintaining concordance with existing signatures, some of which contain an order of magnitude more transcripts. Our findings suggest that novel gene signature holds promise for refining early-stage LUAD prognostic modeling and informing treatment strategies.

**Supplementary Information:**

The online version contains supplementary material available at 10.1038/s41598-025-18567-w.

## Introduction

Lung and bronchus cancers continue to outpace other cancers in projected cancer deaths in recent years^1^, with an estimated 127,070 Americans expected to succumb to lung cancer annually. This figure nearly matches the combined death toll for the next three most common cancers: colon, pancreatic and breast. Approximately 85% of lung cancer diagnoses are of the non-small cell lung (NSCLC) variety^2^. NSCLC is further categorized into three main subtypes: lung adenocarcinomas (LUADs, 40–50% of lung cancers), squamous cell carcinomas (20–25% of lung cancers), and large cell carcinomas (10–15% of lung cancers), of which LUAD is the most prominent subtype, even in non-smokers (< 100 cigarettes)^3^. Lung cancer exhibits extensive heterogeneity, and a higher tumor burden compared to many other cancer types. Among NSCLC subtypes, LUADs harbor more therapeutically actionable aberrations^4,5^. Coupled with the clinical effectiveness of targeted therapies in LUAD, there has been a notable shift towards a driver oncogenic paradigm in the treatment of NSCLC^6,7^.

In recent years, the advent of updated tyrosine kinase inhibitors (TKIs) targeting *EGFR* and *ALK* alterations combined with the introduction of PD1 and PD-L1 inhibitors in 2015, has progressed the development of therapies for advanced and metastatic LUAD. However, earlier stage LUAD has seen few recent advances in therapy^8^. As many patients do not present with symptoms at early-stage, vigilance for early detection is key^9^. For those with high risk of lung cancer, a low dose computed tomography screening program augmented with risk prediction models may serve as the best course of action for overall mortality reduction and population-wide early detection^10^. The stagnation of progress in identifying early-stage treatments for LUAD patients highlights the need for an in-depth systems-level analysis of the complex genetic and molecular interactions that allow for the initiation and progression of LUAD tumor biology in earlier stages (stages 1 and 2).

Prevalent practice involves the use of TNM staging to inform patient prognosis; however, relying solely on anatomical factors alone may not accurately predict patient prognosis. Additionally, patients at the same stage may not respond to treatments the same because of patient-specific genetics and tumor heterogeneity. To better understand the role of LUAD tumor biology in earlier stages (where tumor growth is more contained with limited local invasion, reflecting a relatively preserved tissue structure) division of patients into accurate subtypes informing on survival and influencing treatment paradigms is needed^11,12^. Expression-based survival signatures are essential for identifying gene expression markers linked to survival outcomes and have been widely used to predict overall and disease-free survival or stratify NSCLC risk/recurrence^13–21^, but still have limitations in reproducibility and clinical test accuracy. These approaches aid in tailoring therapies to tumor-specific characteristics and are crucial for defining patient subtypes in early-stage LUAD. Most notably, Song^22^, Shedden^23^ and Soltis^24^ identified several molecular subtypes either alone or combined with relevant clinical characteristics, revealing expression signatures that are predictive of patient survival outcomes.

In this study, we applied an integrative systems biology approach to explore a LUAD transcript co-expression network, identifying potential biomarkers linked to patient survival. Differentially expressed gene transcripts, identified as network hubs (top 10% of module members ranked by module eigengene-based connectivity) within five selected modules (those most correlated with overall patient survival and tumor staging), were collectively analyzed using Receiver Operating Characteristic (ROC) curves to enhance survival prediction in LUAD patients. Last, we compared our survival prediction targets to existing LUAD prognostic and survival signatures presented by Song, Shedden, and Soltis^22–24^. Our approach demonstrates a gene combination superior in survival prediction to the comparators, nominating novel prognostic biomarkers underscoring mechanisms underlying LUAD tumor cell heterogeneity, proliferation, metastasis, and patient survival.

## Materials and methods

### RNA-seq data collection and cleaning

TCGA LUAD patient RNA-seq data was acquired from GDC (Genomic Data Commons) via the TCGA data portal: https://portal.gdc.cancer.gov. FPKM values (fragments per kilobase of transcript per million mapped fragments) from the TCGA-LUAD project were downloaded and log2 transformed for each participant along with clinical data totaling 502 primary tumor samples. This log2 transformation was chosen to ensure that the expression data adhere to assumptions of normality and are less susceptible to high dynamic range variances often observed in RNA-seq data. Staging criteria, based on TNM classification, are detailed in Table 1, distinguishing early (stages I–II) from advanced LUAD (stages III–IV). In preparation for ANOVA and parallel bootstrap regression analysis, we first removed samples that did not have staging (n = 8), age (n = 19), sex (n = 0) or t-staging (n = 3) trait data. Age and sex were regressed out during the analysis to account for potential confounding effects, ensuring that survival prediction is based solely on gene expression without demographic biases. We enforced a ≥ 50% missingness restriction by removing transcripts rows with ≥ 50% zero FPKM values. Seven samples with an absolute standardized connectivity more than three standard deviations above the mean were termed outliers and removed from the cohort. Four samples termed “Do not use” by the sample quality GDC whitelist were also removed from the cohort^25^. Patients without clinical data were also excluded. In total, 41 samples and 46,997 transcripts were expunged. Two methods were used to assess the quality of the dataset before and after data cleaning (1) MBatch, a web-based analysis tool (http://bioinformatics.mdanderson.org/tcgabatcheffects) and (2) a multidimensional scaling analysis adapting the plotMDS function from the R statistical program package limma (Supplementary Fig. 1)^26^. An overview of patient characteristics, demographic and clinical staging data is provided (Supplementary Tables 1 and 2).

**Table 1:** TCGA clinical trait outcomes and descriptions for 461 primary tumor RNA abundance samples.

Clinical trait	Clinical trait description
Pathologic M	Pathologic M staging. The M category details if there is distant metastasis. Binary category (0,1)
Pathologic N	Pathologic N staging. The N category details if cancer cells have reached nearby lymph nodes. Categorical (0, 1, 2)
Pathologic T	Pathologic T staging. The T category details the size and extent of the primary tumor. Categorical (1, 2, 3, 4)
Stage N0	Pathologic N staging. No regional lymph node metastasis. Binary category (0,1)
Stage N1	Pathologic N staging. Metastasis in ipsilateral peribronchial and/or ipsilateral hilar lymph nodes and intrapulmonary nodes, including involvement by direct extension. Binary category (0,1)
Stage N2	Pathologic N staging. Metastasis in ipsilateral mediastinal and/or subcarinal lymph node(s). Binary category (0,1)
Stage T1	Pathologic T staging. Correspond to lung cancers up to 10 mm (T1a), between 11 and 20 mm (T1b), and between 21 and 30 mm (T1c), respectively. Tumor ≤ 3 cm in greatest dimension, surrounded by lung or visceral pleura, without bronchoscopic evidence of invasion more proximal than the lobar bronchus (i.e., not in the main bronchus). Binary category (0,1)
Stage T2	Pathologic T staging. Tumor > 3 cm but ≤ 5 cm or having any of the following features: involves the main bronchus regardless of distance to the carina, but without involvement of the carina; invades visceral pleura (PL1 or PL2); associated with atelectasis or obstructive pneumonitis that extends to the hilar region, involving part or all of the lung. T2 tumors with these features are classified as T2a if ≤ 4 cm or if the size cannot be determined and T2b if > 4 cm but ≤ 5 cm. Binary category (0,1)
Stage T3	Pathologic T staging. Tumor > 5 cm but ≤ 7 cm in greatest dimension or directly invading any of the following: parietal pleura (PL3), chest wall (Including superior sulcus tumors), phrenic nerve, parietal pericardium; or separate tumor nodule(s) in the same lobe as the primary. Binary category (0,1)
Stage T4	Pathologic T staging. Tumor > 7 cm or tumor of any size invading one or more of the following: diaphragm, mediastinum, heart, great vessels, trachea, recurrent laryngeal nerve, esophagus, vertebral body, or carina; separate tumor nodule(s) in an ipsilateral lobe different from that of the primary. Binary category (0,1)
Pathologic T breakdown	Pathologic T staging. Same T staging values but coded by T- staging subdivisions (e.g. 1, 1a, 1b, 2, 2a, 2b, 3, 4) where each subdivision has a coded number. Categorical (1, 2, 3, 4, 5, 6, 7, 8)
Pathologic T breakdown 3.5	Pathologic T staging. Same T staging values but coded to include stage 3 and 4 into one category (3.5). Categorical (1, 2, 3)
Stage T3.5	Pathologic T staging. Same T staging values but coded to include stage 1, 2, and 3 in one category and stage 3 and 4 into one category. Binary (0, 1)
Pathologic stage	General pathologic staging. Pathologic staging combines the results of both the clinical staging (physical exam, imaging test). Stage group determined from clinical information on the tumor (T), regional node (N) and metastases (M) and by grouping cases with similar prognosis for cancer. Categorical (1, 2, 3, 4)
Pathologic Stage 3.5	General pathologic staging. Same as pathologic stage but coded to include stage 3 and 4 into one category (3.5) Categorical (1, 2, 3)
Stage 1	General pathologic staging. Usually, a small cancer that has yet to spread to any lymph nodes and is > 4 cm in size. Binary (0, 1)
Stage 2	General pathologic staging. Usually, a tumor that is ≥ 5 cm in size and may or may not have spread to close lymph nodes. Could be larger ≤ 5cm in size but has not spread to lymph nodes. Binary (0, 1)
Stage 3	General pathologic staging. Generally increased size of tumor, possible tumor invasion of other structures in the lung, spread to lymph nodes and typically cannot be fully resected (may require chemo/radiation). Binary (0, 1)
Stage 4	General pathologic staging. Cancer has spread to another tissue outside of lung or fluid outside of lung. Subdivision of stage 4 based on distant or local metastasis. Binary (0, 1)
Stage 3.5	General pathologic staging. Same as general pathologic stage but coded to include stage 1, 2, and 3 in one category and stage 3 and 4 into one category. Binary (0, 1)
Days to death	Number of days between the date used for index and the date from a person’s date of death represented as a calculated number of days. Continuous (1 … x)
Overall survival (vital status)	Patient vital status (alive or dead). Binary (0, 1)
PFI	Time. Progression Free Interval (PFI) time in days. PFI events are new tumor events like local recurrence, progression of disease, distant metastasis, and new primary tumor of the cancer (tumor event type = NA cases are included) or died with the cancer without new tumor event
PFI censored	Time. Progression Free Interval (PFI). PFI events are progression of disease, local recurrence, distant metastasis, new primary tumors all sites, or died with the cancer without new tumor event. This variable only includes the time in days for patients that had a PFI event (time until next noted cancer event)
PFS	Time. Progression Free Survival (PFS). PFS events are progression of disease, local recurrence, distant metastasis, new primary tumors all sites, or died from any other causes (tumor event type = NA cases are included). This variable only includes the time in days for patients that had a PFS event
PFS censored	Time. Progression Free Survival (PFS). PFS events are progression of disease, local recurrence, distant metastasis, new primary tumors all sites, or died from any other causes (tumor event type = NA cases are included). This variable only includes the time in days for patients that had a PFS event
DSS	Time. Disease Specific Survival (DSS) in days. DSS events are patients who likely died from tumor (for cause of death, or tumor status = with tumor with vital status = dead)
DSS censored	Time. Disease Specific Survival (DSS). DSS events are patients who likely died from tumor (for cause of death, or tumor status = with tumor with vital status = dead). This variable only includes the time in days for patients that had a DSS event
DFI	Time. Disease Free Interval (DFI) in days. DFI events are new tumor events like local recurrence, distant metastasis, and new primary tumor of the cancer (tumor event type = NA cases are included) after complete remission/response is noted in the clinical documentation. If not available then R0 in residual tumor, negative in margin status are assessed
DFI censored	Time. Disease Free Interval (DFI) in days. DFI events are new tumor events like local recurrence, distant metastasis, and new primary tumor of the cancer (tumor event type = NA cases are included) after complete remission/response is noted in the clinical documentation. If not available then R0 in residual tumor, negative in margin status are assessed. Were DFI was 1 for new DFI event censored otherwise

### Network construction

Key modules were identified through correlation with all available LUAD patient traits. Co-expression network analysis was performed via the clustering method WGCNA v1.70.3 (Weighted Gene Correlation Network Analysis) R package^27^. 13,486 gene rows and 461 samples were used for network construction. The ideal soft threshold for the scale-free topology and adjacency calculation was determined graphically (β = 10.2; scale-free fit > 0.8; mean connectivity = 48.9). For automatic network construction, module detection relied on the blockwiseModules function, used with detailed specifications shown in Supplementary Table 3. Genes that were not correlated to a module of at least 100 gene products were assigned the color grey (not in a module). Significance of module correlation to traits was defined by a student’s p-value ≤ 0.05 for biweight midcorrelation (bicor) of the first principal component of a module (module eigengene, ME) to each trait encoded numerically either as a continuous or binary variable, with bicor from − 1 to + 1 as a heatmap scale (Supplementary Fig. 2). Bicor was used instead of Pearson correlation to calculate robust correlations while reducing susceptibility to outliers^28^. Bicor and associated Student p-values are ideal for summarizing correlation robustly because RNA-seq data regularly has high dynamic range and variance across samples. To ensure robustness, we confirmed that DESeq2^29^ normalized counts and FPKM-based networks were similar via module preservation analysis and by Fisher’s exact test for overrepresentation of count-based modules into the FPKM network modules. Differentially expressed (see below Sect. 2.4) hub genes, defined as the top 10% by connectivity within key modules, and anti-correlated modules were prioritized based on module membership (kME) scores and associations with key clinical traits (stage and survival), to identify those most biologically relevant to LUAD outcomes.

### Gene ontology enrichment analysis

Gene ontology enrichment analysis was performed by GOparallel (https://github.com/edammer/GOparallel), on WGCNA module member gene symbols. BaderLab’s monthly updated .GMT formatted ontology gene lists, and additional curated pathways from molecular signature MSig^30,31^ C2 database, Reactome^32^ and WikiPathways^33^ were used as references to determine the association of network modules with assembled lists focusing broadly on regulatory target gene sets, chemical and genetic perturbations, and canonical pathways. Module member gene lists were tested for statistical overrepresentation in select biological processes, molecular functions, and cellular components. Specifically, one tailed-Fisher’s exact test (FET) for hypergeometric overlap was used to assess significant enrichment of module members (gene names) with genes in each GO term, determining if the observed overlap is greater than expected by chance. Additionally, module-specific gene lists were subjected to the same hypergeometric overlap test against experimentally confirmed target genes for selected miRNA cancer driver candidates^34–36^. All reported Fisher exact p-values are adjusted to false discovery rate (FDR) by the Benjamini–Hochberg method to ensure robust significance levels (see Supplemental Materials).

### Differential expression analysis

To identify significant differences in gene expression across tumor stages, we conducted a one-way ANOVA on log2-transformed RNA-seq data, enabling the testing of the null hypothesis that mean gene expression is equal across stages 1 (n = 246), 2 (n = 115), 3 (n = 78), and 4 (n = 22). Differential expression analyses included pairwise comparisons between these stages to highlight key gene expression changes relevant to disease progression. Comparisons were conducted between adjacent and non-adjacent stages (e.g., stage 1 vs. stage 2, stage 2 vs. stage 3, stage 1 vs. stage 4, stage 1 vs all other stages) to capture a comprehensive view of gene expression dynamics.

Separate subgroup analyses within each stage were performed to account for variability among clinical groups. Following the ANOVA, Tukey’s Honest Significant Difference (HSD) post-hoc test was applied for pairwise stage comparisons to maintain control over family-wise error rates. Pairwise comparisons, equivalent to t-tests when conducted post-ANOVA, were adjusted using the Benjamini-Hochberg (BH) correction to manage the false discovery rate (FDR) across all genes.

Significance thresholds for differential expression were set at a p-value ≤ 0.05 and a log2 fold change (L2FC) cutoff of |0.5|, selected to capture biologically meaningful expression changes. These criteria were chosen to balance the detection of subtle but potentially significant biological changes with a conservative FDR limit. The ANOVA pipeline, including gene-specific analysis, FDR adjustment, and L2FC computation, was automated to standardize comparisons across groups. The 8 genes making up the reported LUAD prognostic signature were tested for significance not only as above, but also in the DESeq2 framework using downloaded counts and a FDR-corrected Wald test.

### ROC testing of combinatorial transcript normalized abundance ratios as predictors of survival

In our survival analysis, we focused on overall survival (OS), defined as the duration from diagnosis to death from any cause, with censoring for patients who were alive at their last recorded follow-up. OS data and survival status were obtained from Liu^37^, who provided the detailed data structure used in this study. Specifically, death events are coded as 1 for deceased patients and 0 for those alive, while OS.time represents survival duration in days, calculated as the maximum of either the last contact date or the death date (information is detailed in Table 1). This structured approach provides a standardized and clinically relevant endpoint, effectively capturing disease progression and survival outcomes in LUAD.

To evaluate the predictive potential of combinatorial transcript ratios for LUAD patient survival, we conducted a three-step normalization of gene-level data using CLUSTER 3.0 (http://bonsai.hgc.jp/~mdehoon/software/cluster/command.txt) as described in Karn^38^. Normalized expression levels for differentially expressed and hub transcripts (considered as the top 10 percent of module members (i.e., transcripts) ranked by kME) were summed and ratioed in combinations of 1:1, 2:2, 3:3 or 4:4. Prognostic ratios of sums were calculated for all samples. Each high-expression/high-survival candidate gene’s normalized abundance (sum) was divided by within-sample low-expression/high-survival gene abundance (sum), therefore opposing alterations in sample would amplify sensitivity for survival prediction and ROC specificity if the expression patterns were net coordinated within and across samples, thereby enhancing correlation of the transcript abundance combination to survival measure(s). Corrected and complete OS for the TCGA LUAD cases by Liu^37^, was used to inform linear model fits of each set of ratio calculations (prognostic predictor) to survival outcomes at 5 survival time points (12 months, 18 months, 3 years, 5 years and 10 years)^37^. The R glm function, with binomial variance family function and default linkage (binomial), was used to assess prediction accuracy. The pROC R package was utilized to test and plot each ROC curve using a binary OS time-dependent value and ordered glm-fit predictor. Area under the curve (AUC) was used to rank the top predictor ratios for specific time points and as an average AUC for the 5 time points. AUC 95% confidence interval (CI) calculations were made using the R verification package function ci. As previously described in Dill^39^ iterative assembly of combinations of gene transcript normalized abundance ratios was performed with transcripts nominated via our pipeline, with initial nomination focusing on transcripts that are deemed differentially expressed between stage 1 and stage 2 comparison and present as hub genes from anticorrelated modules in the network. Pipeline-nominated transcript lists from M3-like and M6-like modules assembled into prognostic indicator ratios were tested and by three subsequent rounds of ROC analysis as described in Dill^39^ with noted distinctions. Additional nomination tactics (e.g., literature, pathway databases, the OncoScore package and top survival-associated miRNAs) were used for additional rounds ROC curve analysis, attempting to achieve higher predictive performance (i.e., AUC) and are included in the supplemental materials.

### Comparison of seminal survival related signatures and our top-performing survival signature

To evaluate the performance of our 8-gene survival prediction signature relative to existing LUAD survival-related signatures (e.g. Song, Shedden, and Soltis signatures), we conducted a systematic analysis that included data normalization, prognostic ratio calculations, ROC analysis, and heatmap visualization of correlations. A Research Compendium (RC) supporting this analysis, was developed to best adhere to FAIR (Findability, Accessibility, Interoperability, and Reuse of digital assets) principles^40^, includes all code and data and, is available online (https://github.com/cdyoung2/LUAD_Signature).

For each LUAD sample, we calculated prognostic ratios using gene-specific abundances derived from each signature. Prognostic ratios were calculated as the sum of expression for high-expression/high-survival genes divided by the sum of low-expression/high-survival genes within each sample. This ratio-based approach amplifies the survival prediction sensitivity by leveraging coordinated expression patterns within each signature. To assess the predictive accuracy of each signature at multiple survival intervals, we conducted ROC analysis using our five survival time points. ROC curves were generated using the pROC package in R, and the AUC was calculated at each time point as a measure of predictive accuracy. We then compared AUCs across the signatures using DeLong’s test, a non-parametric approach to evaluate whether differences in AUC values were statistically significant.

To visualize the associations between gene signatures and survival-related modules, we generated heatmaps representing bicor correlations and their statistical significance. Each heatmap displays the strength and direction of the correlation between each gene signature and specific LUAD-associated biological modules, with particular emphasis on the M6 and M3 modules due to their established relevance to survival outcomes. The bicor (biweight midcorrelation) method was applied to compute robust correlations between gene signatures and survival-related modules. This method, known for its resistance to the influence of outliers, was chosen to accommodate the high dynamic range and variance typical of RNA-seq data. Statistical significance for each bicor was evaluated as a Student’s p-value, with a significance threshold of p ≤ 0.05, enabling the identification and ranking of modules significantly associated with each survival signature. The bicors were visualized as a heatmap using the WGCNA labeledHeatmap function to capture both the magnitude and direction of correlations through color intensity, providing an intuitive, comparative overview of each signature’s alignment with LUAD-associated biological pathways, and enabling for a comprehensive view of the relationships between gene signatures and core LUAD survival-related phenotypes.

### Validation of the 8-gene signature in CPTAC and GSE31210 LUAD datasets

To evaluate the generalizability of the 8-gene survival signature, we conducted external validation using two independent datasets: the CPTAC LUAD RNA-seq dataset (accessed via cBioPortal) and GSE31210, a microarray-based expression dataset profiled using the Affymetrix Human Genome U133 Plus 2.0 platform. This analysis aimed to assess the robustness of our transcriptomic signature across distinct platforms and patient populations.

Both TCGA and CPTAC datasets were processed using a harmonized pipeline to ensure comparability. Briefly, gene identifiers were standardized, genes with > 50% missingness were removed, expression values were log2-transformed (excluding zero values), samples were median-centered, and data were scaled to account for platform-specific differences. CPTAC samples were matched with corresponding clinical outcome data, and 18-month overall survival (OS) was defined as the primary endpoint. We computed ratio-based signatures derived from the current study as well as comparator signatures from Song et al. (2022), Shedden et al. (2008), and Soltis et al. (2023). Predictive performance was assessed using ROC and AUC analyses. All code, data processing scripts, and analysis pipelines are publicly available at https://github.com/cdyoung2/LUAD_Signature.

To validate performance on a different platform, we applied the same analysis framework to GSE31210. Raw series matrix and annotation files were downloaded from GEO. Probe-level expression data were mapped to gene symbols based on the GPL570 annotation platform and collapsed to gene-level by averaging across probes. Normalization followed a three-step CLUSTER 3.0-based approach consistent with prior work: log2 transformation, sample-wise median centering etc. The 8-gene ratio was calculated identically to the TCGA analysis, and ROC analysis was performed using 18-month OS as the endpoint.

We note important differences in platform and cohort composition between datasets. While TCGA and CPTAC employed RNA-seq, GSE31210 was profiled using microarray. Additionally, GSE31210 consists predominantly of early-stage, non-smoking LUAD patients from a Japanese cohort, in contrast to the more heterogeneous U.S.-based cohorts in TCGA and CPTAC, which include a broader distribution of disease stage and smoking status. These biological and technical distinctions may contribute to observed differences in predictive performance and are considered in the interpretation of results.

### Single-cell RNA-seq data processing and visualization

To explore the tumor microenvironmental (TME) context of our 8-gene LUAD signature, we analyzed publicly available single-cell RNA-seq data from GSE123902. Raw dense matrix files from 17 LUAD tumor samples were downloaded and processed using the Seurat v5.0.1 R package. Each matrix was transposed and filtered to create individual Seurat objects using standard quality control filters (min.cells = 3, min.features = 200). Data were normalized, scaled, and reduced using PCA and UMAP. Seurat objects were merged into a global dataset and clustered using a resolution of 0.5. Expression of the LUAD 8-gene signature (ATP6V0E1, SVBP, HSDL1, UBTD1, GNPNAT1, XRCC2, TFAP2A, PPP1R13L) was visualized using DotPlots and FeaturePlots across clusters. Genes not detected in at least two clusters or lacking variability were excluded from violin visualizations. Cluster identities were inferred based on canonical markers from the original study and immune/stromal/tumor annotation patterns.

## Results

### Transcriptomic network associated with LUAD survival and staging

RNA-abundance data from 461 LUAD primary tumors (TCGA) were analyzed for associations with clinical outcomes (Table 1, Supplementary Tables 1, 2). Using WGCNA, 18 modules were identified (Supplementary Fig. 3) and ranked by the number of genes contained within those modules from largest to smallest (M1–M18) (Supplementary Table 4). A comprehensive list of all gene transcripts and module membership via correlations (kME) of each gene transcript to the 18 module eigengenes (MEs, calculated as the first principal component of each module across samples) is provided in Supplementary Table 5. MEs were organized by pairwise correlation as a measure of module relatedness and plotted as a dendrogram and heatmap (Supplementary Fig. 4A). To further validate robustness of our network, we performed a module preservation analysis using normalized counts obtained from DESeq2. Networks from normalized counts closely resembled those from FPKM data (Zsummary > 10, p < 1 × 10^–22^). Overrepresentation analysis confirmed bicor correlations produced similar outcomes, supporting the use of FPKM or DESeq2 normalized data in subsequent analyses.

**Fig. 1:**
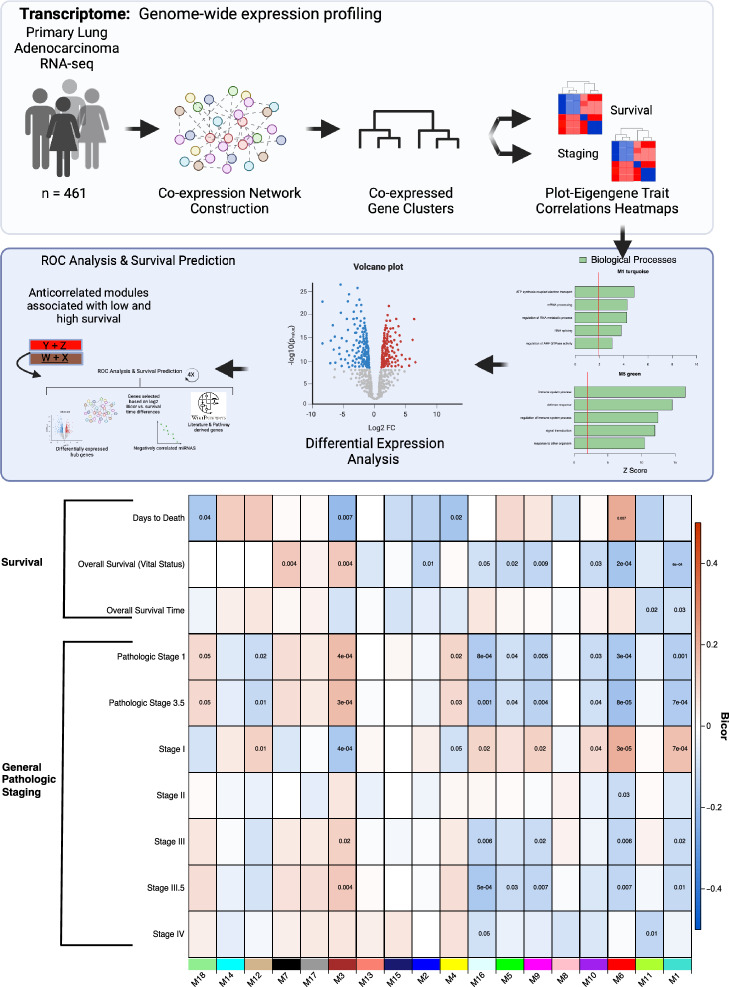
Co-expression network analysis of LUAD transcripts identified modules and module-trait correlations relevant to LUAD. The heatmap displays 18 modules identified. A Kruskal Wallis test among the LUAD case groups with significance set to 0.05, revealed 11 modules were significantly associated with early stage, 7 modules were associated with survival traits, 5 modules (M1, M3, M6, M9, and M16) were associated with both staging and survival (SAS) traits. Overlaid numbers are Student’s p values for bicor significance of trait correlation to the module eigengenes. Module-trait bicor color scale (− 1, blue; 0, white; + 1 red) indicates modules with significant Student’s p cluster together.

To address gaps in early LUAD progression, we focused on modules significantly linked to staging and survival. We identified 11 modules (p ≤ 0.01) correlated with staging traits, either positively (red), or negatively (blue). Furthermore, we identified 7 modules to be significantly correlated with survival traits, and 6 modules (M1, M3, M6, M7, M9 and M11) significantly correlated to both survival and staging traits (Supplementary Fig. 2; Fig. 1). The network identifies survival- and staging-associated modules, revealing two opposing gene co-expression clusters: M1, M6, M9, M16 vs. M3, M7 (Supplementary Table 5). The two module clusters showed an antagonistic expression pattern visualized by robust correlation to multiple early staging traits (Stages: T1, 1, N0, and other binary classifications; Supplementary Figs. 2, 4) as well as their correlations to continuous clinical traits representing survival or tumor progression times. Network inter-module correlation and anticorrelation are further detailed in the correlation heatmap (Supplementary Fig. 4B) and kME table (Supplementary Table 5). Transcripts in the kME table are ordered from most to least representative module members within each module and a green–yellow–red color scale was used to identify positive correlation (red) and negative correlation (green) patterns between modules (highlighting top-ranked hubs best representing each module’s expression pattern). The modules identified with significant stage or survival/progression trait correlation(s) are considered candidate neighborhoods of the gene transcript network, in which exist possible drivers of LUAD initiation or progression, or genes that are signatures correlating to patient survival. However, causation cannot be directly inferred from the network’s undirected co-expression-based connections.

Gene ontology (GO) enrichment analysis assessed the biological coherence of network modules (Top 5 biological processes; Table 2). Key processes included RNA splicing (M1), immune response (M5, M9, M16), lipid metabolism (M6), cell cycle (M3), oxidative phosphorylation (M7), and nucleic acid metabolism (M11). Modules M3 and M7 correlated positively with advanced pathological stage, later-stage disease, and mortality (1 = death), and negatively with early-stage disease.

**Table 2:** Ontology enrichment modules for MEs of interest with correlation to survival or staging clinical traits.

Module		ANOVA p	Top biological processes	GOparallel FET FDR	Block correlation
Turquoise	M1	p = 0.013	RNA splicing	0.03019598	Staging/survival
			mRNA processing	0.03019598	
Blue	M2	NS	Regulation of nucleobase-containing compound metabolic process	5.45E−50	Survival
			Regulation of RNA metabolic process	5.45E−50	
			Regulation of RNA biosynthetic process	4.26E−46	
			Regulation of DNA-templated transcription	8.54E−46	
			Regulation of transcription by RNA polymerase Ii	1.15E−39	
Brown	M3	p = 0.0083	Cell cycle	4.74E−64	Staging/survival
			Cell cycle process	5.98E−60	
			Nucleobase-containing compound metabolic process	1.90E−54	
			Nucleic acid metabolic process	1.94E−53	
			Mitotic cell cycle	7.83E−50	
Yellow	M4	NS	Macromolecule metabolic process	1.11E−10	Survival
			Nucleic acid metabolic process	1.73E−09	
			Primary metabolic process	1.28E−08	
			Nucleobase-containing compound metabolic process	2.89E−08	
			Organic substance metabolic process	7.08E−08	
Green	M5	NS	Immune system process	4.67E−63	Staging
			Defense response	2.73E−61	
			Immune response	1.80E−53	
			Response to external stimulus	4.11E−52	
			Regulation of immune system process	1.18E−49	
Red	M6	p = 0.00039	Lipid metabolic process	3.04E−06	Staging/survival
			Surfactant homeostasis	5.74E−4	
			Chemical homeostasis	1.59E−3	
			Fluid transport	3.78E−3	
			Monoatomic ion transmembrane transport	4.19E−3	
Black	M7	NS	Oxidative phosphorylation	2.30E−35	Staging/survival
			Mitochondrial translation	3.16E−30	
			Aerobic respiration	3.73E−30	
			Cellular respiration	2.24E−29	
			Atp synthesis coupled electron transport	1.90E−28	
Pink	M8	NS	Anatomical structure morphogenesis	1.47E−51	NA
			Extracellular structure organization	5.06E−48	
			External encapsulating structure organization	5.06E−48	
			Extracellular matrix organization	5.06E−48	
			System development	2.44E−43	
Magenta	M9	p = 0.042	Immune system process	6.14E−83	Staging/survival
			Immune response	2.70E−76	
			Regulation of immune system process	1.85E−51	
			Regulation of immune response	3.39E−44	
			Positive regulation of immune system process	8.12E−42	
Purple	M10	NS	Vasculature development	3.03E−32	Staging
			Blood vessel development	6.68E−31	
			System development	2.56E−28	
			Circulatory system development	5.78E−28	
			Anatomical structure morphogenesis	6.22E−28	
Greenyellow	M11	NS	Nucleic acid metabolic process	2.97E−4	Staging/survival
			Regulation of cellular process	5.17E−4	
			Regulation of transcription elongation by Rna polymerase Ii	5.17E−4	
			Rna metabolic process	1.08E−3	
			Regulation of Dna-templated transcription elongation	1.16E−3	
Tan	M12	NS	No significant biological processes associated	NS	Staging
Salmon	M13	NS	Macromolecule metabolic process	4.60E−4	NA
			Protein metabolic process	3.18E−2	
			Protein transport	3.77E−2	
			Primary metabolic process	3.92E−2	
			Organic substance metabolic process	4.76E−2	
Cyan	M14	NS	Cellular respiration	2.59E−3	NA
			Mitochondrial respiratory chain complex assembly	2.59E−3	
			Mitochondrion organization	2.59E−3	
			Generation of precursor metabolites and energy	1.13E−2	
			Aerobic respiration	1.17E−2	
Midnightblue	M15	NS	Rna metabolic process	9.85E−3	NA
			Regulation of nucleobase-containing compound metabolic process	1.44E−2	
			Regulation of Rna metabolic process	1.85E−2	
			Positive regulation of biosynthetic process	1.85E−2	
			Nucleobase-containing compound transport	2.02E−2	
Lightcyan	M16	p = 0.0086	Adaptive immune response	9.87E−157	Staging
			Immune response	1.52E−108	
			Immune system process	5.36E−86	
			Immunoglobulin mediated immune response	3.43E−78	
			B cell mediated immunity	2.09E−77	
Grey60	M17	NS	Cytoplasmic translation	2.61E−120	NA
			Translation	7.12E−85	
			Organonitrogen compound biosynthetic process	1.64E−55	
			Gene expression	1.51E−39	
			Macromolecule biosynthetic process	1.14E−34	
Lightgreen	M18	NS	No significant biological processes associated	NS	Staging

A one-tailed Fisher’s exact test with FDR correction detected significant overlap between GO lists of gene symbols and members of each module. An arbitrary value of ≥ 0.02 was set as criterion for module block-correlation.

### Five modules dually correlated to staging and survival are enriched with differentially expressed genes (DEGs)

**Fig. 2:**
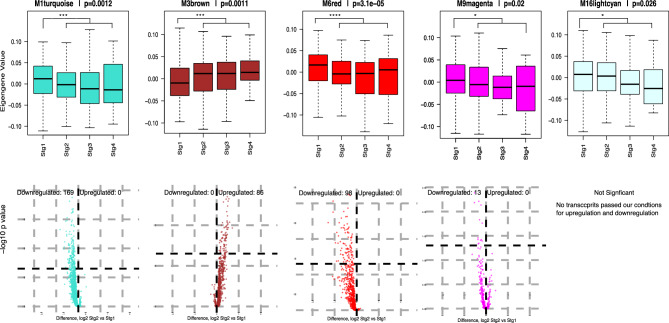
Volcano plots of differentially expressed genes (DEGs) in LUAD transcript cases. **(A)** A one-way ANOVA was used to compare LUAD modules (overall weighted expression patterns) across staging groups. Expression across stages was significant for the 5 SAS-modules (M1, M3, M6, M9 and M16). **(B)** LUAD stage 1 case groups were compared to LUAD later stage case groups for 6 SAS-modules in panel B. Log2 (fold change) for each comparison versus Benjamini–Hochberg FDR is plotted, and gene transcripts are colored by module membership. DEGs were counted for > 50% change (vertical cut-off lines at x =  ± 0.58) and FDR < 5% in each individual comparison. No transcripts passed our conditions for upregulation and downregulation in M16.

**Fig. 3:**
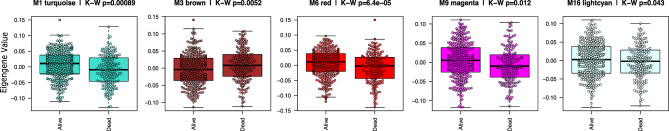
Differences in eigengene values for SAS-modules across LUAD cases. Box plots displaying module eigengene values among the five SAS-modules (M1, M3, M6, M9 and M16) are displayed with Students t-test assessed significant differences between OS(N = 461).

To further elucidate the mechanisms of LUAD initiation and progression, as well as their roles in mortality, we decided to focus on one primary staging clinical trait (pathological staging) and one primary survival trait (OS; binary trait indicating death)) for additional analysis. Pathological staging and OS were selected for their accuracy and clinical relevance in assessing disease extent and treatment impact. Modules M1, M3, M6, M9 and M16, designated as staging and survival (SAS) modules, were significantly correlated with pathologic staging, and were inversely correlated with stage 1 status versus later stages (Fig. 1). We identified M1, M6, M9 and M16 as negatively correlated with increased pathologic staging and OS. Conversely, M3 was positively correlated with increased pathological staging and OS. To explore the strong anticorrelated nature of M3 to M6, M1, M9 and M16 in our SAS-modules, a one-way ANOVA was used to compare LUAD modules (overall weighted expression patterns) across staging groups, and as expected, expression across stages was significant for the SAS-modules (Fig. 2A). Volcano plots (Fig. 2B) show 556 DEGs in stage 1 versus 2, including 336 in our SAS-modules. Eighty-six genes were upregulated (M3) and 280 were downregulated (M1, M6, M9) between the stage 1 and stage 2 groups. Fifty transcripts meeting our criteria for differential expression between stage 1 and stage 2 are detailed in Table 3. Differential expression analysis via DESeq2 confirmed a majority of differentially expressed genes were preserved across both methods. Additionally, a T-test between OS binary outcome groups was used to explore any significant difference between groups that suffered a death event and those that were alive at last follow-up within the SAS-modules. In Fig. 3, SAS-modules are also shown to be significantly differentially expressed between OS groups (boxplots for the other 13 modules are shown in Supplementary Fig. 5 in Supplementary Materials). Modules M1, M6, M9 and M16 all display a similar expression pattern in both OS and pathologic staging, where module gene expression is increased in living and stage 1 samples. Conversely, M3 gene expression detailed decreased gene expression across those same sample groups. Moreover, there was a notable difference in the rate of mortality between stage 1 samples (~ 24%) and later staged samples (~ 50%). To further assess expression changes in early-stage modules, we analyzed DEGs (n = 119, Table 3) between stage 1 and all later stages (Fig. 2A). SAS-modules enriched DEGs were evaluated as possible module driver genes among module transcripts and are bold in Table 3. To further understand the mechanisms in which these possible drivers are modulated we hypothesized that SAS hub + DEGs serve as targets of upstream dysregulated miRNAs, evaluated in the supplemental material results (Supplementary Tables 6, 7, Supplementary Figs. 6 and 7).

**Table 3:** Differentially expressed genes between stage 1 and stage 2 groups (1-way ANOVA) and stage 1 versus all other stages (2-sample T-test) for 5 modules of interest.

Module		DEGs (stage 1 vs. stage 2)	DEGs (stage 1 vs. all other stages)	Up-/down-regulation
Turquoise	M1	hsa-mir-3189, hsa-let-7D, hsa-mir-25, LINC00342, LINC00685, hsa-mir-186, hsa-mir-3682, CLUHP3	hsa-mir-3189, NPIPB15, ZDHHC11B, hsa-let-7D, LINC00342, CLUHP3, LINC00685, hsa-mir-3682	Down-regulated
Brown	M3	FAM83A, **MYBL2, KIF4A**, STC1, **RRM2**, **DEPDC1**, **ANLN**	FAM83A, STC1, **ANLN**, ELOVL6, LYPD3, **RRM2**, SLC2A1, **MYBL2**, **KIF4A**, **CEP55**, TNNT1, **EXO1**, TUBB3, **HMMR**, **DEPDC1**, MKI67, **FOXM1**	Up-regulated
Red	M6	**SCGB3A2**, **CYP4B1**, CRYM, PIGR, WIF1, C4BPA, **CYP2B7P**, **SFTPB**, VSIG2, PRSS12, PLA2G10, **CACNA2D2**, ELAPOR1, DUOXA1, ZBTB7C, ALPL, CLIC6, **HLF**, **FAM189A2**, CYP4X1, DUOX1, **CFAP221**, C5orf49, CEBPA-DT, **B3GNT8**, ENTPD3, SCUBE2, OLFM1, ERICH2, WFDC2, PDZD2	PGC, SFTPC, **SCGB3A2**, **CYP4B1**, CRYM, **CYP2B7P**, AQP5, SCGB3A1, PIGR, C4BPA, SFTPB, **C16orf89**, CLDN18, SFTPA2, DMBT1, **CACNA2D2**, WIF1, PRSS12, **GGTLC1**, SFTPA1, VSIG2, AQP4, SCGB1A1, TMEM130, **HLF**, **LRRK2-DT**, SFTPD, C8orf34-AS1, **ATP13A4**, AGER, FAM189A2, ALPL, ELAPOR1, **SUSD2**, CLIC6, RNASE1, **CFAP221**, CYP4X1, PLA2G10, **NAPSA**, **PEBP4**, **SFTA3**, CEBPA-DT, GGT6, HSD17B6, LMO3, IRX2, TDRD10, **B3GNT8**, **SCTR**, SPINK5, **ESYT3**, PLA2G1B, HABP2, SCUBE2, C5orf49, CFTR, WFDC2, GFRA3, **DAPK2**, MFSD4A, ZBTB7C, **CPAMD8**, FCGBP, ZNF750, KLF15, SUSD4	Down-regulated
Magenta	M9	FDCSP, MS4A1, PTGDS, RAB37	FDCSP, MS4A1, PTGDS, hsa-mir-8071-1	Down-regulated
Lightcyan	M16	No significant DEGs	CD19, CD79A, CR2, IGHJ3P, IGHV3-63, IGLV10-54, IGHD, IGHM, IGHJ1, IGHV3-47, IGLC3, IGKV6D-21, IGKV1D-16, IGLV1-44, IGLC7, IGLV2-18, IGHV3-13, IGKV1-8, IGHV3-64, IGLV3-9, IGLV1-36, IGLV3-27, IGLV8-61	NA

Transcripts in bold are hub genes (top 10% module membership/kME) in modules of interest. Significance was set to p < 0.05 and lfc > |0.5|. Module transcript regulation skewed entirely to up- or down-regulation.

### Survival significance and prognostic implications of anti-correlated modules for LUAD

**Fig. 4:**
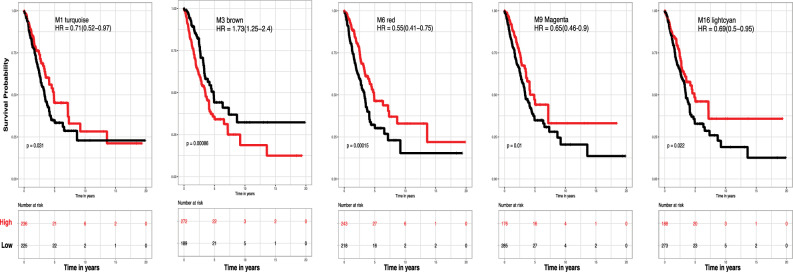
Survival analysis reveals association between SAS-module eigengene values and overall survival in lung cancer. **(A)** Two anti-correlated modules (M3 and M6) driving opposing genetic SAS-module signatures were selected for survival analysis. The M3 module was positively associated with OS and while the M6 module was negatively associated with a death event (OS). Kaplan–Meier (KM) survival analysis significantly distinguished patients separated by high and low module expression in all SAS-modules. The red line in each Kaplan–Meier plot represents survival of cases in the higher expression tier and the black line represents survival in cases with lower expression. A Wald test determined p-values and hazard ratio HR scores with 95% CIs. **(B)** Significantly higher survival with lower expression was seen in all M3 differentially expressed hub genes (MYBL2 p < 0.0001, HR = 2.28, CI = 1.49–2.49; KIF4A p = 0.00085, HR = 1.67, CI = 1.23–2.27; RRM2 p < 0.0001, HR = 2.24, CI = 1.65–3.05; DEPDC1 p < 0.0001, HR = 2.07, CI = 1.49–2.87; ANLN p < 0.0001, HR = 2.52, CI = 1.85–3.42).

Two strongly anti-correlated modules (M6: red and M3: brown) (Supplementary Fig. 4B and Supplementary Table 5) from our opposing SAS-module signatures were selected for further survival analysis. Module M3 correlated positively with a death event (OS) and advanced pathological and clinical stages, while M6 correlated negatively with a death event and later pathological stages but positively with early clinical stage (Fig. 1). Kaplan–Meier (KM) analysis (Fig. 4A) distinguished patients by SAS-module expression. Hazard ratios (HRs) confirmed M3’s opposing HR to M1, M6, M9 & M16 modules. Following M3 module survival association, hub status DEGs which differentiate stage 1 versus stage 2 tumor samples (Table 3) were evaluated via KM analysis (Fig. 4B). Significantly higher survival with lower expression was seen in all M3 differentially expressed hub genes (MYBL2 p < 0.0001, HR = 2.28, CI = 1.49–2.49; KIF4A p = 0.00085, HR = 1.67, CI = 1.23–2.27; RRM2 p < 0.0001, HR = 2.24, CI = 1.65–3.05; DEPDC1 p < 0.0001, HR = 2.07, CI = 1.49–2.87; ANLN p < 0.0001, HR = 2.52, CI = 1.85–3.42).

### Network-nominated ROC testing of multi-gene ratios

We hypothesized that the positive and negative correlation to two key traits and module association with survival candidate genes shown from our previous results could represent genes that interact in determining survival and progression of LUAD patients. To test this hypothesis, we employed ROC curve analysis of ratios of these genes where (1) the numerator genes, when having higher expression, are associated with higher survival and lower stage, and (2) the denominator genes impact survival and reflect the inverse pattern of expression across LUAD tumor stages.

Differentially expressed hub genes (top 10% by kME) from anti-correlated M6 (red) and M3 (brown) modules were selected as LUAD prognostic candidates for the initial round of ROC testing (Table 3; bold transcripts in Stage1-versus-stage2). Given the normalization procedure shown in the methods, and network-defined determination of positions in numerator (high-expression, high-survival) or denominator (low-expression, high-survival). Larger ratios were found to predict better survival in this model.

**Fig. 5:**
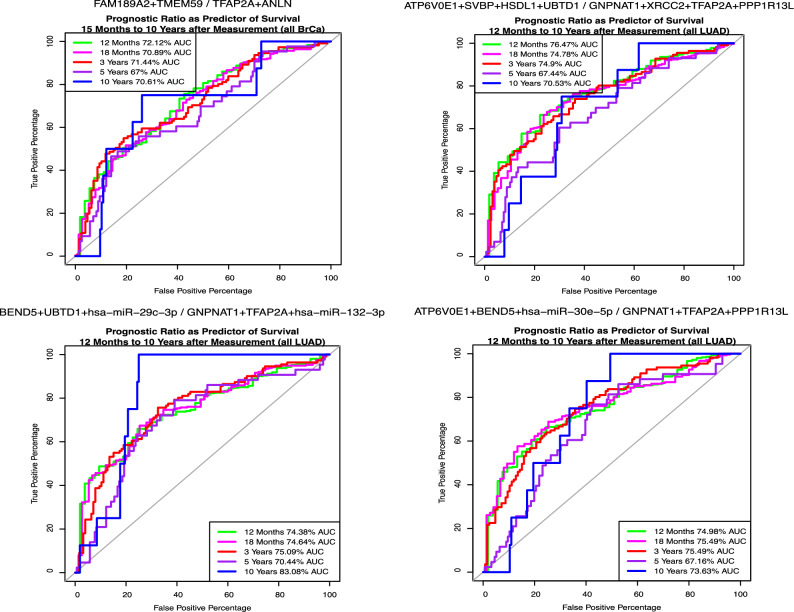
Top performing survival predictors by ROC AUC. ROC curves predicting OS were generated on combinatorial ratios of SAS nominated miRNAs, first using normalized transcript abundances in N = 457 LUAD cases with OS traits. **(A)** In round 1, transcripts consisting of M3 and M6 DEGs and hubs nominated by Kaplan–Meier distinction of OS via their levels in independent RNA-Seq were tested in 6827 combinatorial ratios for OS prediction using all LUAD cases (top performer shown in panel A). **(B)** Round 2 added nominations top OS.time correlated transcripts. **(C)** Round 3 added top OS.time correlated miRNA transcripts **(D)** Round 4 ROC tested whether round 1, + top-performing miRNA nominations from round 3, literature round 2 associated genes, and pathway implicated genes by round 3.

In the initial round of analysis, 6287 relevant differentially expressed gene combinations were evaluated, covering all possible 1:1, 2:2, 3:3 and 4:4 combination prognostic ratios (Supplementary Table 8). All AUCs were > 56% on average across all 5 timepoints, suggesting the selection of hub status DEGs that differentiate stage 1 versus stage 2 tumor samples may impact survival in the expected direction. The best overall mean AUC across all combinations for all timepoints was FAM189A2 + CYP4B1/TFAP2A + ANLN with a maximum AUC of 84.62 at 10 years and 71.67 at 12 months. The top-ranked mean AUC excluding the 10- and 5-year timepoints was 71.48% (FAM189A2 + TMEM59/TFAP2A + ANLN) with the top ratio at 12 months (72.12%; Fig. 5A). The best combination regardless of timepoint was CYP4B1/RRM2 with an AUC of 87.65% at 10 years, although many ratios were similar. In addition, Supplementary Table 8 details ratio associated p-value, CI, accuracy, sensitivity, and specificity.

To improve on our test, we generated a volcano plot using columns B and C in Supplementary Table 9 as our X and Y axis, followed by selection of the extremes in the first and second quadrants. We selected 10 genes from the right extreme (M6) and eight from the left (M3); two genes from round 1 persisted (KIF4A and ANLN). In total, we proceeded with 20 genes, 10 M3-like candidate denominators and 10 M6-like candidate numerators.

A total of 60,626 gene ratios (1:1, 2:2, 3:3, 4:4) were evaluated by ROC AUC (Supplementary Table 10) in Round 2. As expected, prognostic ratios, enriched with OS.time-correlated genes, showed higher AUCs across all time points, averaging > 60%. The top combination, BEND5/GNPNAT1, achieved an 87.58% AUC at 10 years, though statistical power declined due to fewer uncensored samples at this timepoint. The top performing ratio in the cohort excluding the 10-year timepoint (12 months–5 year), ATP6V0E1 + SVBP + HSDL1/CENPE + GNPNAT1 + PPP1R13L (AUC = 76.69%) outperformed the next top ratio ATP6V0E1 + SVBP + HSDL1 + UBTD1/CENPE + GNPNAT1 + TFAP2A + PPP1R13L by only 0.05% and the 150th top AUC by less than 1.0%. Figure 5C illustrates the results of Round 3, in which top OS.time-correlated miRNA transcripts were incorporated into ROC testing, further refining the model’s predictive power. The mean AUC determined top ratio when excluding the 10-year timepoint was ZMYND12 + BEND5 + HSDL1 + UBTD1/GNPNAT1 + TFAP2A + KIF20B + PPP1R13L (all timepoints: AUC = 74.77%; excluding 10-year: AUC = 74.06%). When excluding both the 5 and 10 year timepoint the ATP6V0E1 + SVBP + HSDL1 + UBTD1/GNPNAT1 + XRCC2 + TFAP2A + PPP1R13L ratio assumes the top position with an AUC of 75.54% (Fig. 5B). In an effort to further improve on our prediction, we sorted by 12 month to 3 year and 12 month to 5 year mean AUC and selected the genes that appeared in > 20% of the top 100 performing ratios (most frequently from the numerator: ATP6V0E1, HSDL1, SVBP, UBTD1, BEND5, PEBP1, TMEM59 and denominator: GNPNAT1, TFAP2A, PPP1R13L, ANLN, KIF4A) for another round of ROC AUC analysis communicated in Supplemental Materials.

### Survival-related signature comparison reveals a robust prognostic signature for LUAD

**Fig. 6.**
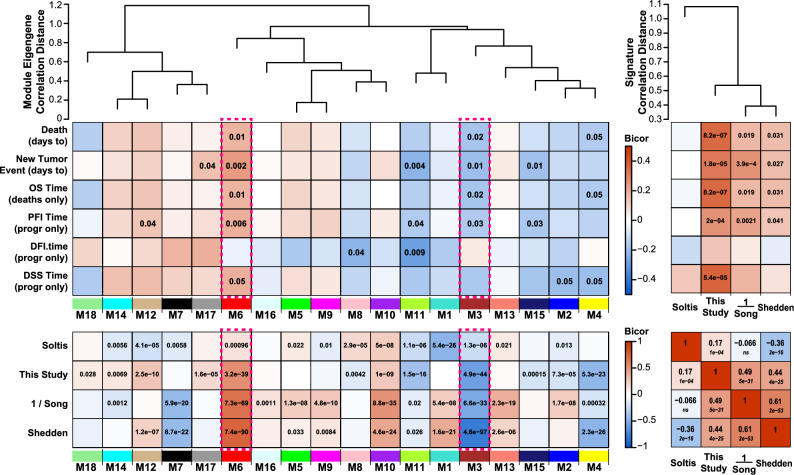
480 LUAD case correlations recalculated to network modules for four curated LUAD prognostic signatures of Soltis, et al., this study, the inverse signature of Song, et al., and the 459 gene signature of Shedden, et al. **(A)** Survival case correlations. MEs were recalculated based on the N = 461 (457 miRNAs matched to 461 transcripts in network) TCGA network in the full 480 LUAD tumor samples curated from GDC (see RC), and their relatedness plotted as a correlation distance dendrogram (top left), MEs recalculated were correlated using bicor to the uncensored quantitative survival (days) traits relating to overall survival (OS), progression free interval (PFI), disease free interval (DFI), and disease specific survival (DSS), as well as days to any new tumor event in patients after biopsy (lower left of panel). Right, the four curated signatures were likewise correlated to the same six survival traits quantified in days. Any significant bicor is annotated with Student’s p value for the correlation over the heatmap. Bicor scale is − 0.5 to + 0.5 for **(A)**. A correlation distance-based dendrogram among the four signatures is displayed at the top right. **(B)** The four prognostic signatures were correlated using bicor to the 18 recalculated module eigengenes (left), and to each other using Pearson correlation (right, Pearson rho displayed as bold text, with Student’s p value below in italics). Any significant bicor (left) is annotated with its corresponding Student’s p value for the correlation over the heatmap. Both bicor and Pearson scales are − 1.0 to + 1.0 for **(B)**. In both panels, key survival modules M3 and M6 are highlighted by a dashed hot pink outline around their survival trait **(A)** and signature **(B)** correlations.

Many seminal survival-related signatures have been developed, but a comprehensive clinical grade survival-related gene signature remains elusive. We asked how the targets predicting survival identified in our study would perform in comparison to existing LUAD prognostic and survival metrics. So, we selected three established signatures^22–24^ for comparison to our round 2 signature calculating each across 480 TCGA LUAD FPKM cases. Recapitulating ME correlations to survival traits (Fig. 6A, *left-panel*), trait correlations were extended to the four signatures for Soltis, the current study, Song, and Shedden studies (Fig. 6A, *right-panel*). Correlation to OS, PFI, and DSS was highly significant for the current study signature, and Song and Shedden signatures also correlated significantly with survival and progression traits. Note M6 and M3, outlined in Fig. 6, are the basis for numerator and denominator genes in the current study’s signature. While our signature correlates with survival traits, we considered all four signatures as additional traits for correlation to the 18 modules (Fig. 6B, *left-panel*). Modules M3 and M6 were the most significant correlates for all signatures except Soltis. Notably, Song’s signature for poor prognosis was inverted to achieve comparable positive correlation considering survival rather than positive correlation to inflammatory response. Interestingly, as modules are ordered by their correlation distance, closest neighbors (M1, M13, M17 and M16) to our modules contributing genes of our prognostic signature (M3 and M6) do not generally correlate in the same direction to signatures (within row). Since M6 and M3 have the highest correlation significance to three of the four signatures, they represent transcripts with a strong and specific relationship to survival. However, the network more generally has most modules correlating significantly to multiple of the four signatures, indicating broad biology captured by these signatures, which are restricted to a small subset of genes. The Soltis’ signature, which was developed to aid in subtype classification of LUAD, has its best module correlation to our M1 SAS-module, with its gene ontology enriching in ATP synthesis coupled electron transport and mRNA processing as top biological processes. This divergence from top modules’ correlation significance of the other three signatures to M6 and M3 likely indicates that the subtype-distinguishing genes profiled in the Soltis signature are less involved with M3 and M6 biological processes like cell cycle and mRNA processing (M3). The Shedden and Song signatures also correlate more broadly to other modules in the same direction as our gene signature, and, they further correlate to each other as a block (Fig. 6B, *right-panel*), suggesting that Song’s inflammatory response genes associate with and predict survival like our signature and Shedden, but the underlying genes in the signatures are largely different, with our signature’s eight genes overlapping with none of the genes in the other three signatures. Indeed, Shedden and Song signatures correlate well to this study’s signature (0.44 and 0.49), having been generated and tested independently in different LUAD data. Remarkably, the current study round 2 optimized signature, comprising 8 unique and novel genes not present in the Shedden (459 genes), Soltis (155 genes), or Song (11 genes) signatures, compares favorably as a survival signature to these established models.

We validated survival prediction via gene expression ratios by first testing TCGA-LUAD normalized expression data against disease-specific survival (DSS), defined as death with concurrent “with tumor” status or disease-specific mortality. ROC analysis of the top-performing 8-gene ratio predictor (Fig. 5D), using DSS instead of OS, demonstrated similar performance at the 18-month timepoint. The gene ratio identified from LUAD network analysis predicted both OS and DSS, supporting its reproducibility and clinical relevance as a survival predictor in LUAD patients. To further assess generalizability, we performed cross-dataset validation using two independent cohorts. In the CPTAC LUAD RNA-seq dataset (accessed via cBioPortal), predictive performance for 18-month survival remained robust (AUC = 70.59%), closely matching TCGA (AUC = 70.84%), further confirming reproducibility. In GSE31210, a microarray-based dataset, the 8-gene ratio achieved moderate predictive accuracy (AUC = 64.52%), supporting the signature’s cross-platform applicability despite expected variation due to differences in data generation, platform, processing, and cohort composition.

### Single-cell RNA sequencing of gene signature expression across LUAD tumor microenvironment

**Fig. 7:**
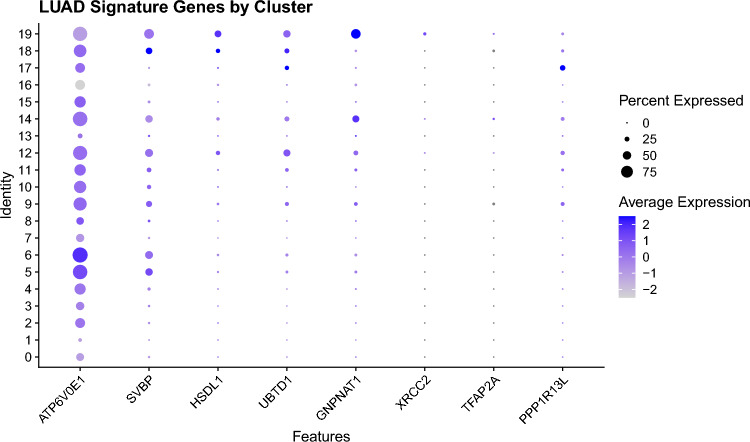
Dot plot summarizing the expression of the eight-gene LUAD survival signature across annotated cell clusters in single-cell RNA sequencing data from 17 LUAD tumors (GSE123902). Dot size indicates the proportion of cells within a cluster expressing the gene (percent expressed), and dot color reflects scaled average expression. TFAP2A, GNPNAT1, HSDL1, UBTD1, and SVBP show selective expression in epithelial and stromal clusters, while PPP1R13L and XRCC2 are more broadly distributed. ATP6V0E1 exhibits relatively low expression overall. These data highlight the potential contribution of both tumor-intrinsic and microenvironmental compartments to the signature. A UMAP visualization of cell clustering and identity annotations is provided in Supplementary Fig. 8.

Following quality control and preprocessing, UMAP dimensionality reduction and clustering identified diverse cell populations within the tumor, including putative epithelial, immune, and stromal compartments in GSE123902. Expression of the 8 signature genes was assessed across clusters and individual samples. Of the eight genes, five (ATP6V0E1, GNPNAT1, HSDL1, TFAP2A, and UBTD1) showed detectable expression in multiple clusters, while three genes (PPP1R13L, SVBP, and XRCC2) exhibited sparse or undetectable expression across samples. UMAP feature plots revealed the spatial distribution of signature gene expression across individual cells, highlighting cluster-specific localization (Supplementary Fig. 8). Dot plot visualization summarized the average expression and percent-positive cells for each gene by cluster (Fig. 7). Together, these findings suggest that the 8-gene signature likely reflects contributions from multiple cell types within the LUAD microenvironment, including tumor epithelial and immune compartments.

## Discussion

Well-executed transcript co-expression networks provide a systems biology framework of modules which have coherent biology inherent in their organization. They can be correlated to disease or sample-specific traits with little concern for false positive correlation. This permits biological insight into the complex network of molecules, while further helping researchers nominate novel biomarkers of disease, drug targets, and mechanistically important key drivers of disease processes^41,42^. Systems biology approaches, such as WGCNA can help untangle the biological complexity of the LUAD malignant state. While LUAD has been well-studied, the complex interactions of mRNA and their influence on survival are not well understood. This study produced a systems-view of the LUAD transcript network as a resource, and we demonstrate a use case in which we nominate influential candidate gene combinations of mRNA able to nominally predict survival (mRNA/miRNA combinations; supplemental materials). Furthermore, our mRNA signature demonstrates its competitiveness by comparing favorably to well-established mRNA signatures, with some comprising over 50-fold as many genes.

Co-expression analysis revealed 18 modules, many of which correlated with key clinical outcomes. Transcripts correlated across multiple samples are the basis of co-expression networks; those genes that are tightly interconnected (e.g., high module membership, kME) have similar expression patterns, and upstream of this shared expression pattern, one can infer common transcription factors, miRNAs or epigenetic mechanisms^43^. Seven of 18 modules correlated with our selected traits of interest (pathological staging and OS). Six had negative correlation (M16, M5, M9, M10, M6, M1) and one (M3), positive correlation (Fig. 1). Five of the seven modules were identified as strongly significant across both OS and pathological staging and termed SAS-modules. SAS-modules were significant for several biological processes as mentioned previously (Table 2), namely cell cycle and DNA metabolic related processes (M3), immune activation (M9 and M16), and solute transport across membranes and molecular metabolism processes (M6 and M1).

Our results suggest two competing gene expression patterns distinguishing early from later-stage LUAD. Early-stage patients exhibit elevated expression of genes involved in RNA processing, surfactant homeostasis, chemical homeostasis, and innate immunity (M1, M5, M9, M6, M11), which decline with advancing stage. The association of cell cycle activity with worsening stage and survival supports the well-established role of growth-promoting alterations in tumor progression. Additionally, increased metabolic activity linked to excretion and fluid transport (M6), positively correlated with lower stage and improved survival, highlights early immune system activation in lung cancer initiation^44^. Two SAS-modules (M3 and M6) were revealed to be anti-correlated across OS and staging. We therefore termed modules of transcripts that have low-expression and are associated with high-survival M3-like, and those with high-expression associated with high-survival as M6-like (M1, M6, M9, M16). SAS-module genes with hub and DEG status are considered vital drivers of early LUAD. We hypothesized that SAS-modules, positively correlated to later-stage clinical traits and negatively correlated with longer survival time, are enriched with suitable targets for potential therapeutic correction. SAS-modules could potentially be regulated via miRNA-mediated downregulation by significantly negatively correlated miRNAs, as mentioned in the supplementary materials.

In an effort to add in possible causality and directionality we included expression of upstream regulators of mRNAs where possible to perform ROC analysis, with AUC evaluation of predictor strength as described in Dill^39^. We began with a logical selection of transcripts that were both DEGs and hub genes of M3(-like) and M6(-like) modules (round 1) and evolved our original selection of transcripts by selecting genes significantly correlated to OS.time (round 2). Round 1 gene selection was validated by > 55% AUC across all timepoints. The top ranked ratio nominated by AUC for round 2 (Supplementary Table 10) included genes, ATP6V0E1, GNPNAT1, TFAP2A and PPP1R13L that were maintained in top-ranked ratio AUCs throughout subsequent rounds of ROC analysis (genes SVBP, UBTD1 and XRCC2 did not persist through subsequent rounds). In round 2, our top ratio selected by mean-AUC across all timepoints, ATP6V0E1 + SVBP + HSDL1 + UBTD1/GNPNAT1 + XRCC2 + TFAP2A + PPP1R13L (Fig. 5B) contained a known driver of lysosome and phagosome maturation in LUAD cells^45^, ATP6V0E1. TFEB, a known regulator of ATP6V0E1 was also selected for round 4 ROC (Supplemental Materials) testing but was not present in our top genes ranked by mean AUC.

Conversely, our denominator contains two recognized autophagy-related genes, TFAP2A and PPP1R13L^46–49^. Reports on TFAP2A’s effects on tumorgenicity have been contradictory. In some cancers, TFAP2A expression acts like a tumor suppressor, while in others it has tumorigenic potential^50^. In our review of the literature, TFAP2A in LUAD leans towards tumor-promotion via numerous recorded mechanisms^51–54^. PPP1R13L/iASPP is a well-known oncogene, but recent studies have indicated it may have an proapoptotic role via its interaction with NF-κBp65^54^. In our study, PPP1R13L was routinely enriched in the low-expression high-survival group of our ratios suggesting more than likely, an oncogenic function in LUAD. PPP1R13L/iASPP is a well-known oncogene, but recent studies have indicated it may have an proapoptotic role via its NF-κBp65 interaction^54^. Presence of active p53 is a driver of PPP1R13L expression and regulation and suggests why a later well-performing nominated gene, GADD45GIP1, detailed in the supplemental materials, a binding partner of all GADD45 isoforms, is routinely enriched in top predictor gene combinations^55,56^. Interestingly, a ratio containing only known interactors PPP1R13L and GADD45GIP1 did not surpass 60% AUC at any time-point, suggesting that other genes were additive. Multiple reports have identified GNPNAT1, a key enzyme in the hexosamine biosynthetic pathway (HSB) as a prognostic marker in LUAD^57,58^. HSB intermediates and the end-product UDP-GlcNAc serve as key substrates in many biochemical pathways, allow for crosstalk across many tumor promoting processes, and may serve as a sensor of energy availability^59^. Of the remaining genes in our signature, XRCC2, a key DNA repair gene in the homologous recombination pathway, is overexpressed in LUAD and promotes tumor cell migration and invasion via vimentin stabilization. Elevated XRCC2 expression is associated with poorer overall survival, supporting its role as both a prognostic marker and potential therapeutic target in LUAD^60^. SVBP, though less studied in LUAD, encodes a protein involved in tubulin detyrosination, a post-translational modification critical for microtubule stability^61,62^. Aberrant tubulin dynamics have been linked to chemoresistance and metastatic potential in NSCLC, and SVBP was consistently expressed at lower levels in high-survival ratios in our analysis. HSDL1, a peroxisomal enzyme involved in lipid metabolism, has been implicated in the immunological and metabolic landscape of LUAD. One study identified HSDL1 as part of a lipid metabolism–related survival signature, with elevated expression associated with poorer prognosis in LUAD patients^63,64^. Additionally, HSDL1 expression negatively correlates with anti-tumor immune infiltration and positively with tumor-promoting immune cell types^64,65^. Lastly, UBTD1, a ubiquitin-like protein that interacts with MDM2 to stabilize p53, also regulates TGF-β signaling and mechanotransduction pathways. In LUAD, reduced UBTD1 expression promotes tumor aggressiveness via RhoA activation and epithelial-to-mesenchymal transition (EMT), suggesting its function as a tumor suppressor may be context-dependent^66^. Top ranked mean combinatorial ratio AUCs did not reach clinical test accuracy (85% +) but were considered fair predictors of survival at all timepoints. ROC analysis provided an avenue for assessment of additive or synergistic transcript level contributions to OS, and its prediction in LUAD. Further investigation into the network module member genes’ intra- and inter-module connectivity may highlight relevant biology for LUAD initiation, progression and module association to survival and staging traits. Additionally, while clinical application requires further refinement, this gene signature could inform personalized treatment strategies by guiding the selection of chemotherapeutic regimens or adjusting treatment intensity based on prognosis, potentially improving outcomes for patients with poorer prognoses. Our correlation analysis provides a bridge between our knowledge of LuCa literature and current survival signatures, to showcase an integrated approach to understanding LUAD. Additionally, it may offer insights into future studies, to understand more deeply the divergent performance of these signature correlation patterns and their variation in ROC-AUC. An interesting observation from the correlation heatmap (Fig. 6) is that M3 brown and M6 red modules are highly negative or positively correlated for three of the four signatures. The differences between these signatures can be attributed to the differences in their design. These variations stem from their deviation in distinct conceptual frameworks, that are formed from slightly different predictive factors and gene selection or retention criteria. One such difference is the anticorrelation of the Song signature (best visualized directly from the RC code, but visible here as opposing red/blue for most modules). This should be attributable to an opposing outcome that the signature is intended to predict, most likely severity rather than survival. Song’s signature anticorrelation to the others might reflect it’s intended use to predict poor prognosis and drug therapy response prediction, and not survival, as death or progression is the flip side of the same coin.

Recalculations in 480 TCGA cases provide some replication and enable quantitative benchmarking against the Song, Shedden, and Soltis signatures. Our signature correlates most strongly with Song, followed by Shedden, with Soltis being a distant third. Although not specifically selected for, our findings reinforce the link between immune-related genes (IRGs) and LUAD prognosis and survival—two related but distinct attributes. Notably, three of our signature genes (ATP6V0E1, TFAP2A via GSDMD interaction, and PP1R13L) have documented roles in the inflammatory response.

In addition to tumor-intrinsic gene expression programs, recent studies underscore the importance of the TME in shaping disease progression and therapeutic response in early-stage LUAD. Immune-suppressive or immune-excluded TMEs—characterized by stromal remodeling, regulatory immune populations, and impaired CD8⁺ T-cell infiltration—are associated with poor prognosis and resistance to immunotherapy^55,67–69^. To contextualize our 8-gene survival signature within this framework, we assessed cell-type–specific expression of the signature genes using single-cell RNA-seq data from LUAD tumors (GSE123902). Five genes (TFAP2A, GNPNAT1, HSDL1, UBTD1, SVBP) were detectably expressed in specific epithelial or stromal-immune clusters, suggesting the signature reflects both tumor-intrinsic and microenvironmental features.

This interpretation is supported by prior literature: TFAP2A activates ITGB4, promoting epithelial growth and suppressing CD4⁺/CD8⁺ T-cell infiltration via NF-κB; PPP1R13L (iASPP) modulates NF-κB and mTOR signaling with implications for tumor–immune crosstalk; GNPNAT1 correlates with immune infiltration metrics and poor prognosis in LUAD; and ATP6V0E1, a V-ATPase subunit, contributes to acidification of the TME, which can impair immune activity and facilitate metastasis. These data suggest our transcript signature may capture both oncogenic signaling and microenvironmental interactions that jointly influence LUAD survival.

Collectively, these findings align with emerging models of LUAD pathogenesis in which tumor-intrinsic transcriptional programs interact with the TME to shape disease progression and prognosis. Our validation in two independent cohorts—CPTAC (RNA-seq) and GSE31210 (microarray)—further supports the cross-platform and cross-cohort robustness of the 8-gene signature. While microarray data inherently contains less missingness but greater measurement variability, the 8-gene signature retained predictive performance across this platform, supporting its robustness. These results highlight the potential utility of ratio-based transcriptomic predictors that integrate signals across cellular compartments and may serve as scalable biomarkers for early-stage LUAD risk stratification. However, these insights must be interpreted in the context of study limitations, as outlined below.

### Limitations

Our identification of candidate biomarker ratios from gene expression sets has limitations. While the best ratio AUCs exceed 97% at the 10-year timepoint (Round 2), they do not surpass 70% at earlier timepoints. Notably, 95% confidence intervals suggest that the low number of observations at 10 years does not impact power. ROC curve performance may be constrained by compensatory changes in co-expressed genes, which modulate rather than directly influence survival. Additionally, OS.time correlations with gene expression were limited to ± 0.3 for mRNAs, suggesting that current ratio AUCs may be near their maximum achievable values.

Furthermore, TCGA tumor profiles represent pre-treatment status, limiting our ability to capture post-treatment transcriptomic changes that may affect therapy response or survival interpretation. Additionally, candidate selection in was constrained by the computational demands of unbiased ROC testing, with Round 4 alone generating over 200,000 combinations from 17 numerator and 13 denominator candidates. Machine learning could enhance transcript and combination selection, enabling more complex, weighted ratios and prioritizing OS-time correlated genes across diverse pathways. LUAD cases in TCGA may also exhibit greater heterogeneity than breast cancer subtypes like triple-negative breast cancer, which Dill^39^. used for survival prediction modeling. Lastly, validation was limited by the small number of TCGA-LUAD cases with paired miRNA and mRNA data, necessitating an alternative survival metric. The recalculation method used here is adaptable to other datasets and may facilitate module reproduction for comparative analyses.

In conclusion, this study utilized a network-based approach to identify stage- and survival-associated biomarker genes influencing LUAD patient outcomes. Our integrative systems-biology framework provides a foundation for further exploration of LUAD initiation and progression. Pilot analyses of the network reveal that transcript modules targeted by miRNAs do not simply follow expected feed-forward regulation but instead exhibit complex interactions. Our optimized systems analysis pipeline, detailed in the supplemental materials, serves as a resource for the LUAD research community by refining prognostic gene and miRNA combinations. The top-performing combinations involve lysosomal and autophagic processes, the hexosamine biosynthesis pathway, an apoptosis inhibitor, and the pro-oncogenic transcription factor TFAP2A. While these genes are well-established in LUAD, their combinatorial power for survival prediction suggests potential synergistic interactions that warrant further investigation. Comparative analysis underscores our signature’s robustness, demonstrating stronger correlations with survival traits than existing signatures while relying on fewer genes, enhancing clinical utility. Further exploration of intra- and inter-module connectivity may refine a prognostic signature and uncover additional biological insights for LUAD.

## Supplementary Information

Supplementary Table 1.

Supplementary Table 2.

Supplementary Table 3.

Supplementary Table 4.

Supplementary Table 5.

Supplementary Table 6.

Supplementary Table 7.

Supplementary Table 8.

Supplementary Table 9.

Supplementary Table 10.

Supplementary Information.

## Data Availability

The datasets analyzed for this study can be found in the TCGA Data portal: https://portal.gdc.cancer.gov.
