# Supplementary material for: Integrated systems biology reveals an 8-gene signature predicting early-stage lung adenocarcinoma progression and patient survival
Source: Sci Rep. 2025 Oct 6;15:34762. doi: 10.1038/s41598-025-18567-w (PMC12501061; doi:10.1038/s41598-025-18567-w)
Supplement: Supplementary file 11 — Supplementary Information. [file 41598_2025_18567_MOESM11_ESM.docx]

Supplementary Material

# Supplementary Methods

**miRNA integration into ME and nomination of miRNA cancer driver candidates**

To further test mRNA-miRNA influence on cancer driving genes, a total of 662 batch-effect normalized miRNAs abundances were obtained from the Pan-Cancer Atlas (<https://gdc.cancer.gov/about-data/publications/pancanatlas>) and matched to 457 of 461 cases in the LUAD transcript network. RNA-Seq, clinical and normalized miRNA data were matched across TCGA sample IDs followed by log_2_ transformation of abundance. For miRNA integration into the co-expression network, the WGCNA bicorAndPvalue function was used to calculate student correlation p-values for biweight midcorrelations of each miRNA to each transcript network ME, a sample-wise set of module eigengene values representing the weighted within-module pattern of member transcripts, equal to their first principal component. Bicor and Student’s p-values were generated for each ME across all TCGA sample IDs for all miRNAs. The top five most negatively correlated and highly significant miRNAs (inclusion criteria: p-value ≤ 10^-10^ and bicor ≤ -0.3) were selected as candidate miRNA drivers for further analysis. In total, 41 miRNA LUAD driver candidates were selected. MiRNAs were subset into two categories, bottleneck miRNAs, miRNAs that passed inclusion criteria for more than one co-expression module and non-bottleneck miRNAs, miRNAs that passed inclusion criteria for only one co-expression module (Supplemental Table 6).

**miRNA mapping to targets – identification of key driver miRNAs & miRNA targets**

To further explore miRNA target mapping, MiRNet merges and integrates target and interaction data from 14 miRNA databases (TarBase, miRTarBase, miRecords, miRanda (*S. mansoni* only), miR2Disease, HMDD, PhenomiR, SM2miR, PharmacomiR, EpimiR, starBase, TransmiR, ADmiRE, and TAM 2.0) into a “miRNA interaction knowledgebase” and provides a functional web-based graphical user interface [22, 23]. The web application (<https://www.mirnet.ca/miRNet/home.xhtml>) miRNet was used to collect the list of experimentally validated targets of our 41 miRNAs of interest (last access date: 12/27/21. Supplemental Table 7). miRNA-gene interactions were sourced from miRTarBase v8.0 and TarBase v8.0 [24, 25]. The manually curated and experimentally validated miRNA-target gene interactions were initially visualized in miRNet. Hub gene transcripts targeted by miRNAs were screened and cross-referenced with module-specific over-represented gene lists. Hub genes were considered as the top 10% of transcriptome module members ranked by kME.

# Supplementary Results

**SAS modules integrate with strongly anticorrelated miRNAs**

Integration of miRNAs into the LUAD network was performed by calculating student’s correlation p-values for biweight midcorrelations across miRNA abundances available in the TCGA Pan-Cancer Atlas (457 paired case measure). The top five significant negatively correlated miRNAs to each of our SAS modules are shown in Supplemental Figure 6. To get gross separation of case-specific measurements based on segregation of miRNA abundance, tertiles were defined by ME relative abundances across samples. A Student’s t-test was conducted to find significance of differences between upper- and lower- tertile miRNA abundance, where tertiles were defined by ranked transcriptome network eigengene values. Tertiles for the top negatively correlated SAS module miRNAs are provided in Supplemental Figure 7. We choose a tertile split to have a clear distinction between high- and low-level abundance miRNA groups. MiRNAs were further restricted with inclusion criteria based on correlation rho ≤ -0.3 and significance ≤ 10^-10^. A list of 41 possible upstream post-transcriptional regulator candidate module driver miRNAs remained (Supplemental Table 6). Ten of the 41 candidates negatively correlated with multiple modules, consistent with being bottleneck miRNAs. We chose to build Supplemental Table 6 as a framework and resource for further analysis into the complex biology of LUAD and the role miRNAs may play in post-transcriptional regulation of the LUAD molecular network.

We hypothesized that some candidate driver miRNAs are not only upstream of the modules they regulate but also may serve as possible re-enforcers of SAS traits, either by acting on many transcripts within a module, acting on key driver hubs of modules or targeting major driver kinases in LUAD initiation and progression that lie within these modules. To test this hypothesis, module hubs were evaluated for enrichment with experimentally validated targets of their respective candidate regulator. FDR of hypergeometric overlap was calculated for both hubs and the total member list for all miRNAs significantly negatively correlated to modules in the LUAD network (Supplemental Table 6). Total module gene lists and module hubs among the top 10 percent of genes ranked by intramodular kME (correlation rho to the first principal component of their module using bicor) are enriched for known experimentally confirmed target genes for our candidate miRNAs (Supplemental Table 6 and 7). Interestingly, hsa-mir-335-5p, the miRNA that reaches significant overlap for the most modules (6 modules: M2, M5, M6, M8, M9 and M10), is also significantly negatively correlated to M11 (Supplemental Table 6, Supplemental Figure 4**)** indicating that this miRNAs’ target mRNAs are not generally enriched inside the co-expression module to which the miRNAs best negatively correlate, fitting with a more complex biological picture than we hypothesized. No other miRNA reached five modules of significant overlap, so for the purpose of further investigation, we focus on bottleneck miRNAs, as they may have the most widespread impact across the LUAD transcriptomic network. Moreover, targets of these bottleneck miRNAs such as CENPEN, ZEB2, and BIRC5 have key roles in LUAD biology [39-41].

**Additional ROC testing of combinatorial transcript normalized abundance**

To improve predictive performance we sought to include miRNA abundances into our ROC AUC analysis, following a 3-step normalization process outlined in Karn, et al [30] were we where able integrate normalized, negatively correlated, miRNA abundances into our expression data set and elect miRNAs based on two different selection criteria: (1) a round 1-like nomination criteria (volcano plot generated using miRNA OS.time correlation and associated p-values) and (2) miRNAs based on association with LUAD patient survival in the literature, bottleneck miRNA status, and/or election via OncoScore (Supplemental Table 11).

MiRNAs selected for integration into ROC testing round 3 via criteria 1 were miRNAs present in the extremes and near extremes on the right and left of the volcano plot (not shown). We selected 17 total miRNAs, 8 from quadrant one and nine from quadrant two. Only 1 of the top OS.time correlated genes were present in our 41-miRNA candidate list (hsa-miR-31-5p; non-bottleneck, negatively correlated with M6 red module). Additionally, 14 of 17 miRNAs had OncoScores above the OncoScore optimal cut-off value of 21.09, indicating known association of these miRNAs to cancer (Supplemental Table 11). Out of our 41 identified miRNA candidates, 19 miRNAs, 17 of which had miRNAs with OncoScores above the optimal cut-off value (21.09) or were bottleneck miRNAs were selected. Addition of miRNAs reduced mean AUC values across all timepoints (Supplemental Table 12 and 13). Mean AUC for all ratios was previously >60% in round 2 analysis but 26,736 of 175386 ratios fell below 60% (criteria 1 selection), and 76625 of 169729 (criteria 2 selection) in subsequent round 3 analysis (Supplemental Tables 12 & 13). Additionally, these ratios were enriched with miRNA-containing combinations. Top mean AUC for round 3 criteria 1 and criteria 2 were BEND5+UBTD1+hsa-miR-29c-3p / GNPNAT1+TFAP2A+hsa-miR-132-3p (75.53%) and ATP6V0E1+BEND5+HSDL1 / GNPNAT1+TFAP2A+ANLN (74.40%) respectfully. Mean AUC across all timepoints was the only metric in which round 3 AUCs outperformed round 2 AUCs. Top performing ratios from both rounds contained BEND5+UBTD1 in the numerator and GNPNAT1+TFAP2A in the denominator suggesting a key role of these transcripts in LUAD patient survival (Figure 5C). BEND5, a transcriptional repressor and known tumor suppressor gene. Reduced BEND5 expression has been associated with advanced stage and worse prognosis in BrCa patients, and its hypermethylation promotes cell growth in colorectal cancer patients [43, 44]. Functionally, BEND5 can inhibit BrCa metastasis and growth in vivo and in vitro via interruption of Notch signaling [43]. A final transcript among M6-like genes was a miRNA nominated in round 3 (criteria 1), hsa-miR-30e-5p. Hsa-miR-30e-5p is a known NSCLC tumor suppressor frequently shown to be downregulated. Hsa-miR-30e-5p has been suggested to negatively regulate cancer stem cell marker USP22s’ oncogenic effects via SIRT1/JAK/STAT3 signaling [45].

In our final round of ROC testing (Supplemental Table 14), we integrated literature- and pathway database-derived genes resulting from a systematic review. Ultimately, we added 10 logical literature and pathway associated genes to round one 11 frequency-selected genes for further evaluation in our third round of ROC AUC testing. We nominated hub gene ATAT1, a tubulin acetyltransferase and VASH1, a tyrosine carboxypeptidase implicated by SVBP’s role in post-translational modification of tubulin [42, 43]. A hub gene, GADD45GIP1, involved in growth arrest via negative regulation of cyclin dependent kinases was implicated by PPP1R13L’s role as tumor suppressor in non-transformed cells [44]. Additionally, NKIRAS1, a NF-kappa-B inhibitor gene similar to PPP1R13L was also included to see if NFKB inhibition was a key pathway implicated in top performing gene combinations. A G protein-coupled receptor kinase ADRBK2/GRK3 was also nominated because of its involvement with numerous carcinogenic processes [45, 46] and upregulated expression in LUAD by TFAP2A [47]. CITED2 was also nominated as a known TFAP2 co-activator that functions as a molecular switch of proliferation induced by cytokines [48, 49]. Hub genes CDK1 and PLK1 were nominated through a GNPNAT1 literature-association search in which CDK1 and PLK1 were two of five significantly highly expressed kinases in tumor tissues, significantly related to OS and co-expressed with GNPNAT1 in LUAD [50, 51]. Hub gene HMMR was implicated via its high co-expression to GNPNAT1 and differential expression across multiple comparisons in our analysis [50]. Last, ATP6V0E1, a lysosomal gene, that encodes an enzyme that hydrolyzes ATP and aids in translocation of protons across the plasma membrane, has been associated with LUAD [52] and implicates the MiT/TFE transcription factor TFEB, which promotes ATP6V0E1 and autophagosome and lysosomal gene expression [53]. Additionally, we also elected to keep 8 top ranked miRNAs across multiple measurements (AUC across all timepoints, excluding 10-year and excluding 10- and 5-year mean AUCs) as they were enriched in some of our highest performing ratios in round 3 (hsa-miR-374a-5p, hsa-miR-29c-3p, hsa-miR-30e-5p, hsa-miR-22-5p, hsa-miR-140-3p, hsa-miR-92a-3p, hsa-miR-132-3p, and hsa-miR-1293).

In total, 205,310 combinations spanning 1:1, 2:2 and 3:3 gene ratios were evaluated by ROC AUC using literature and pathway database suggested interactions. AUC best performers surpassed 90% at the 10-year timepoint but were poor discriminators at earlier time points (range: 57.72%-67.66%). Interestingly, nomination of literature- and pathway database-derived genes did not meaningfully increase mean AUC across 12 months to 3-year (75.32% vs 75.54% in round 2), 12 month to 5 year (73.85% vs 74.06% in round 2), and 12 month to 10 year (75.53% vs 74.77% in round 2) calculations. Round 2 nominated genes were primarily enriched among top performing AUCs, indicating our selection of literature and pathway associated genes did not markedly increase survival prediction across timepoints (Supplemental Table 14). The top-ranked ratio by mean AUC across all timepoints for round 4 is shown in Figure 5D.

**Further evaluation of Signature Comparisons**

As mentioned in Dill, *et al*, [36] pipeline iterations were denoted from literature, pathway databases and the OncoScore package and top survival-associated miRNAs [30, 37] for additional ROC curve analysis’ attempting to achieve higher predictive performance (i.e. AUC). Signed summed calculation versus equal weight calculation comparison of Song, Shedden Soltis and Young signatures, with a correlation distance (n=480). Initially, the objective was to compare the signature we developed with three current and well-received signatures. The Research Compendium (RC) runs the above signatures through our pipeline (FPKMs or COUNTs) and outputs prognostic ratio ROC-AUC curves for comparison to ours. The ROC-AUC graphs measure performance, and this final step assesses the degree of correlation among these signatures. We added module relatedness (Pearson correlation distance) dendrogram to show how relatedness of modules may affect correlation direction of the neighboring modules. Module relatedness (different from your figure) now represents the correlation structure and relatedness of module eigengenes considering all samples available. It is important to note that following assignment of the 99xx rows of cleanDat to module colors, each of the eigengenes is calculated de novo as the 1st PC of all genes assigned to a color. These eigengenes incorporate network module's gene members (98.6%), the network definition which benefitted from removal of outliers and noisy (low-expressed) genes.

**Supplementary Figures and Tables**

**Supplementary Figure 1.** MDS Plots for data cleaning. A principal component analysis was performed, adapting the plotMDS function for TCGA-LUAD data prior to data cleaning and outlier removal (panel A) and after data cleaning and removal of low network connectivity outliers (panel B). Changes in the range of the axis post filtering, outlier removal, and transformation were minimal, and batches represented by different colors in panels A and B are not tightly clustered, indicating variation due to batch effect was also minimal. Samples colored by module color (ME1-ME18).

**Supplementary Figure 2.** **Full Co-expression Heatmap.** Co-expression network analysis of LUAD transcripts identified modules and module-trait correlations relevant to LUAD. The heatmap displays 18 modules identified. An overlaid numbers are Student's p values for bicor significance of trait correlation to the module eigengenes. Module-trait bicor color scale (-0.5, blue; 0, white; +0.5 red) indicates modules with significant Student’s p cluster together.

**Supplementary Figure 3. Dendrogram of 18 modules in expression data.** Cluster dendrogram of 18 modules detected in TCGA-LUAD gene expression data. Module color assignments are detailed in the first row labeled Module Colors. Genes not co-expressed in any modules are assigned to the grey module. Positive (red) and negative (blue) correlation for all coded traits are detailed in the row below.

**Supplementary Figure 4.** **Eigengene Network.** Module relatedness clustering dendrogram and heatmap of 18 modules detected via WGCNA. (A) Cluster dendrogram of MEs based on ME correlation. (B) Heatmap of 18 MEs organized by module related order.

**Supplementary Figure 5.** **Box plots of significant OS binary groups for all MEs not including SAS-modules.** Box plots detailing significant difference in OS binary groups for all MEs, not including SAS modules are displayed. There is a significant difference between high and low expression for ME7, ME2, ME5, and ME10. A one-step calculation of Student correlation p-values for multiple biweight midcorrelation was determined.

**Supplementary Figure 6. Scatterplots.** Fischer exact test heat map displays overlap of miRNA targets with each module. Mir335-5p reaches significant overlap for two of the five SAS modules plus four additional modules, M2, M5, M8, and M10. FDR corrected p-values are reported.

**Supplementary Figure 7. Boxplots of miRNAs for SAS-modules.** A tertile split between low and high abundance miRNA groups for the SAS modules. Significance of differences between log2 transformed upper- and lower- tertile miRNA abundance was determined using a Student’s t-test, with tertiles defined by ranked transcriptome network eigengene values.

**Supplementary Figure 8. Full Gene-by-Sample UMAP Layout.** UMAP-based feature plots for each of the 17 signature genes across single-cell RNA-seq profiles from valid LUAD samples. Each panel displays gene expression scaled per cell. Sample metadata and clustering are consistent with prior dot plot representations.


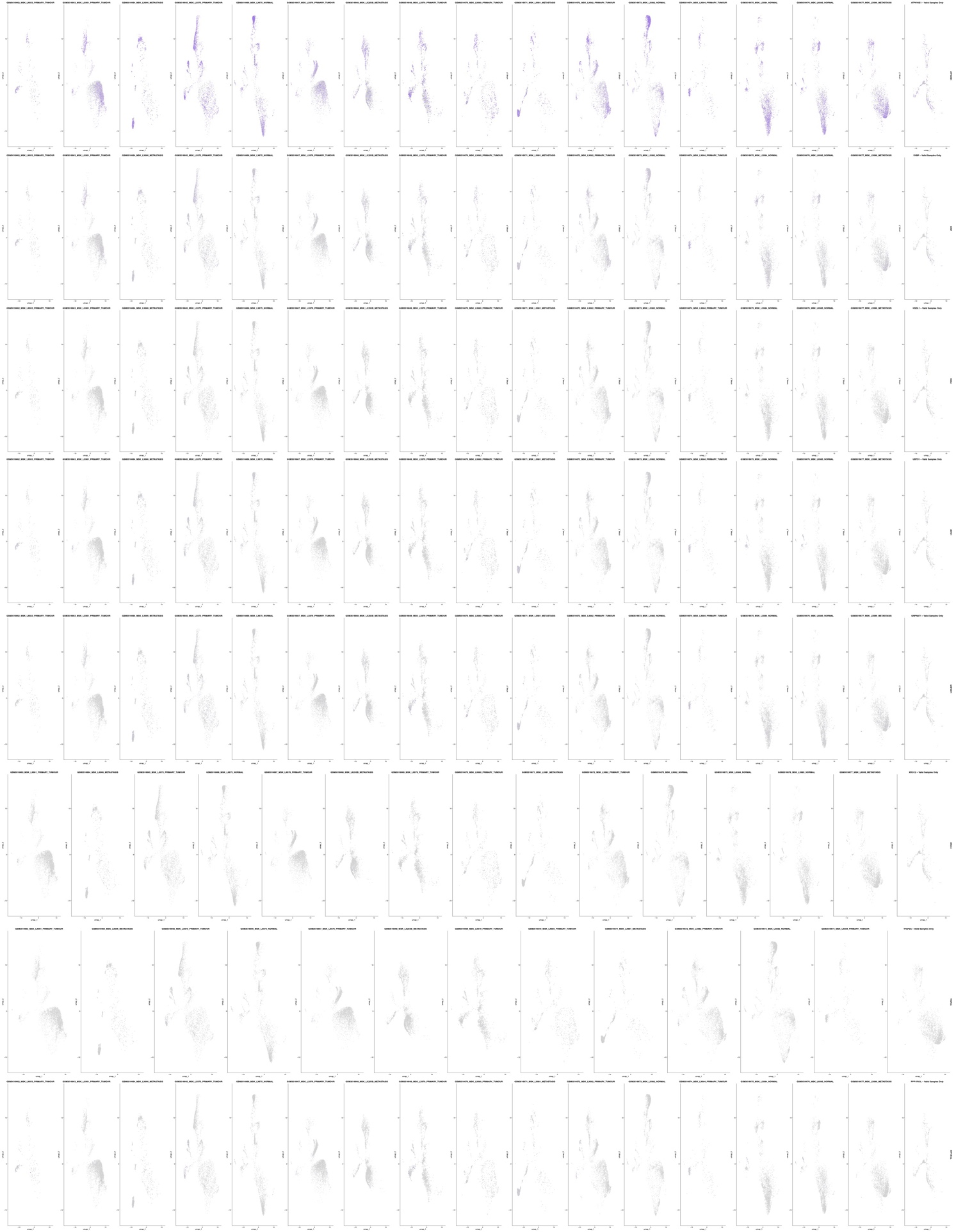


**GitHub Repository Link**

https://github.com/cdyoung2/LUAD_Signature
